# Effects of Food Art Therapy on the Self-Esteem, Self-Expression, and Social Skills of Persons with Mental Illness in Community Rehabilitation Facilities

**DOI:** 10.3390/healthcare8040428

**Published:** 2020-10-24

**Authors:** Ju-Hye Kim, Kwisoon Choe, Kyoungsook Lee

**Affiliations:** Department of Nursing, Chung-Ang University, Seoul 06974, Korea; jessie001@hanmail.net (J.-H.K.); kyoungsooklee0628@gmail.com (K.L.)

**Keywords:** self-expression, mental health, rehabilitation, self-esteem, social skills

## Abstract

Persons with mental illness often have low self-esteem, a lack of self-expression, and poor social skills. This study used a quasi-experimental two-group pre-test-post-test design to investigate the effects of food art therapy on the self-esteem, self-expression, and social skills of persons with mental illness attending community rehabilitation facilities. The authors recruited persons with mental illness aged 18 years or older attending three community rehabilitation facilities. Participants in two rehabilitation facilities participated in food art therapy (experimental groups 1 and 2; *n* = 15 for each group), and participants in the third rehabilitation facility participated in regular programs of the facility (control group, *n* = 30). Participants in the experimental groups attended a total of eight sessions of food art therapy twice per week for four weeks. The Korean versions of the Rosenberg self-esteem scale, self-expression scale, and social skill rating system were administered at pre- and post-test in both the experimental and control groups. The self-esteem, self-expression, and social skills of the experimental group improved significantly compared to the control group. The findings suggested that food art therapy would be an excellent psychosocial intervention to help persons with mental illness to rehabilitate in the community.

## 1. Introduction

For a long time, people have formed a sense of community by cooking, eating, and sharing food [1]. While cooking together, people learn cooperation, such as respecting others by making food using natural ingredients [2]. Thus, people experience psychological healing by focusing on cooking activities [1,3]. People also feel emotional stability while cooking [4]. Therefore, the authors designed this study to determine if people with mental illness in the community experience psychological healing from cooking.

Food art therapy is a form of psychotherapy first developed in Korea [5] that is not yet well known outside of Korea. Specifically, food art therapy enables people to express their inner world through creative play and artistic activities using food ingredients, which leads to positive thinking and self-discovery [5]. By gathering in a small group and sharing stories about a particular theme through food ingredients, individuals experience an improvement in their self-expression and sociality [6], as well as in their self-esteem [7]. For example, children who participate in a food art therapy program significantly improve their self-expression and sociality more than children who do not participate [6]. Among young adults, female college students who participate in food art therapy show improved self-esteem [7].

Food art therapy may help people with mental illness, who generally have low self-esteem and difficulty adjusting to society and establishing interpersonal relationships [8,9]. Self-esteem refers to evaluating one’s abilities and values, and people with high self-esteem look at themselves positively [10]. Additionally, self-esteem is related to a sense of accomplishment, psychological satisfaction, and good interpersonal relationships [11]. When self-esteem increases, psychiatric symptoms and anxiety decrease [12]. Therefore, self-esteem is a critical therapeutic factor for people with mental illness [10]. People can improve their self-esteem through practice and growing accustomed to an activity, even if they are initially unskilled [13]. Activities in food art therapy require no special education or talent. People can gain a sense of accomplishment and satisfaction quickly through cooking activities, thereby improving their self-esteem [2,14]. One hypothesis of this study was that food art therapy improves the self-esteem of people with mental illness.

Individuals are also able to express themselves in food art therapy. Self-expression is an interpersonal skill that selects appropriate actions and verbal expressions to communicate one’s feelings honestly and clearly without violating the human rights of others [15]. Food art therapy provides people with a means of self-expression using ingredients [6,16]. Therefore, another hypothesis of this study was that food art therapy increases the self-expression of people with mental illness.

Food art therapy has increased the sociality of people [16]. In particular, cooking activities engaged in by people in similar situations promote their social skills while allowing them to share information and experiences [17]. The third hypothesis of this study was that food art therapy improves the social skills of people with mental illness. To date, few studies have examined the effectiveness of food art therapy in people with mental illness. This study aimed to verify the effects of food art therapy on the self-esteem, self-expression, and social skills of people with mental illness who attend community rehabilitation facilities.

## 2. Materials and Methods

### 2.1. Design

This quasi-experimental study sought to identify the effects of food art therapy on the self-esteem, self-expression, and social skills of people with mental illness in community rehabilitation centers.

### 2.2. Participants

**Figure 1:**
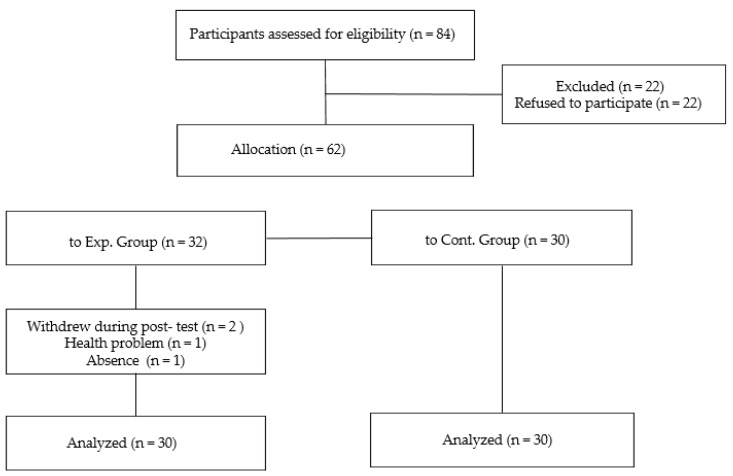
Flow chart of participants’ inclusion.

All subjects gave their informed consent for inclusion before they participated in the study. The study was conducted in accordance with the Declaration of Helsinki and was approved by the Institutional Review Board of the Chung-Ang University (1041078-201906-ZZHR-200-01). The authors recruited persons with mental illness who met the inclusion criteria at three mental health centers in South Korea. The inclusion criteria were: (1) aged between 18 and 60 years, (2) diagnosed with mental illness according to the Diagnostic Statistical Manual-IV diagnostic criteria, (3) living in the community and attending community psychiatric rehabilitation facilities, and (4) a global assessment of functioning (GAF) score ≥ 51. The exclusion criteria were as follows: (1) having organic brain syndrome or intellectual disability and (2) having difficulty cooking as judged by staff at the facilities (Figure 1).

All participants were informed of the purpose and method of the study and of their right to withdraw at any time. Data collection was conducted after the participants voluntarily signed the informed consent form.

### 2.3. Measures

#### 2.3.1. Rosenberg Self-Esteem Scale

The Korean version of the Rosenberg self-esteem scale [18] was used to measure self-esteem. The scale consists of 10 questions with a 4-point Likert-type scale: five questions are related to positive self-esteem, and five questions are related to negative self-esteem. Examples of positive items are “On the whole, I am satisfied with myself” and ”I take a positive attitude toward myself”. Negative items are “At times, I think that I am no good at all” and “I certainly feel useless at times”. This scale has been scored on a metric ranging from 10 (poor) to 40 (excellent). The higher the score, the higher the self-esteem. In this study, Cronbach’s alpha coefficient was 0.85. The item-total correlation for both positive and negative items of the Rosenberg self-esteem scale is 0.4 or higher.

#### 2.3.2. Self-Expression Scale

The self-expression scale [19] was used to measure the self-expression ability of participants. It comprises three sub-scales (contents, verbal expression, and non-verbal expression) and consists of 20 questions on a 5-point Likert scale, in which the higher the score, the greater the self-expression ability. Examples of items are “The contents of the conversation are not clear”(contents), “I hesitate before speaking”(verbal expression), and “When I speak, I cannot make eye contact with the other person”(non-verbal expression). Cronbach’s alpha coefficient in this study was 0.85. The Cronbach alpha coefficient for the subscales of the self-expression scale was 0.37 (contents), 0.83 (verbal expression), and 0.74 (non-verbal expression), respectively.

#### 2.3.3. Social Skill Rating System

The Korean version of Gresham and Elliott’s (1990) [20] social skill rating system [21] was used to measure social skills. This scale comprises three subscales: cooperation, assertion, and self-control, and consists of 30 questions with a 3-point Likert scale. The higher the score, the better the social skills. Examples of items are “I use my time appropriately while waiting for someone else’s help” (cooperation), “I can say compliments to a colleague” (assertion), and “I am very patient with disagreements with colleagues” (self-control). In this study, Cronbach’s alpha coefficient was 0.84. The Cronbach alpha coefficient for the subscales of the social skill rating system was 0.60 (cooperation), 0.86 (assertion), and 0.53 (self-control), respectively.

### 2.4. Data Collection

#### 2.4.1. Food Art Therapy Program

Based on a literature review of food art therapy and discussions with mental health professionals of the community rehabilitation facilities, the authors structured the preliminary food art therapy program. Two certified food art therapists reviewed the contents and structure of the preliminary food art therapy, and the overview of the final program is presented in Table 1.

#### 2.4.2. Implementing a Food Art Therapy Program

Persons with mental illness of three mental rehabilitation facilities participated in this study. Thirty participants from mental rehabilitation facilities A (*n* = 15) and B (*n* = 15) participated in the experimental group, and 30 participants from mental rehabilitation facility C participated in the control group. Participants in the control group participated in regular programs of the facility. Examples of the regular programs are social skills training, sports, such as table tennis, reading books, and walking. Participants in the control group were given gifts in return for participation in the survey. One of the authors (J.K.), who is a certified food art therapist, executed the program at mental rehabilitation facilities A and B during eight bi-weekly sessions from September 2 to 30 (experimental group 1) and October 7 to 31 (experimental group 2) in 2019. Each session lasted an average of 80–90 min.

Of the eight sessions, the program focused on building intimacy in the first two sessions and on knowing and thinking deeply about oneself in sessions 3–4. Sessions 5–6 focused on expressing oneself to others, and the seventh and eighth sessions focused on encouraging and praising others and forming a positive image of the future.

Each session began with a greeting and a brief discussion about the weather, followed by the introduction of the topic of the day. A worksheet was prepared to provide participants an opportunity to think more deeply about the questions related to the topic for the day’s food art activity. For example, worksheet questions for the topic of the third session activity included: what I want to hear the most; write your three wishes and three words that you want to hear the most on a pancake and decorate it; introduce the reasons and think about yourself, and express it. After taking the time to complete and share the worksheets, participants started the food art activities. Each participant did their work using the given ingredients and then explained the finished work to others. When the participants described their work, we recorded their voices but did not analyze the contents thematically. Examples of what the participants said are as follows. In Session 3, “The word that I want to hear the most”, some participants shared the following stories: “I was so happy and happy that others told me what I wanted to hear”, “I think my wish has already come true. I feel strong and thought I could do it too”. In Session 5, “Making important people”, the participants said: “If I go home today, I will tell my family that I love you”, “I want to see my family as I am making a family. It touches me that there are family members.”

### 2.5. Data Analysis

Data were analyzed using SPSS version 23.0 (IBM Corp., Armonk, NY, USA). Descriptive statistics were calculated to examine the main characteristics of the participants. As a result of the Kolmogorov–Smirnov and Shapiro–Wilk test, the normality of self-esteem, self-expression, and social skills was all confirmed. Thus, the non-parametric test was not performed. We used the independent sample t-test, chi-square test, and Fisher’s exact test to test the homogeneity of the experimental and control groups. Paired sample t-test and independent-sample t-test were used to compare the measures before and after the intervention to test its effects on self-esteem, self-expression, and social skills. All statistical tests were set at the 0.05 significance level, with a 95% confidence interval. Internal consistency reliability was assessed using Cronbach’s alpha.

## 3. Results

### 3.1. Homogeneity of Demographic Characteristics between the Groups

The demographic characteristics of the participants and the homogeneity test on the demographic characteristics are presented in Table 2. There were no statistically significant differences in the demographic characteristics, thereby indicating sufficient homogeneity.

### 3.2. Homogeneity of Self-Esteem, Self-Expression, and Social Skills between the Groups

Regarding subjective self-esteem, mean scores for the experimental and control groups were 22.90 ± 5.06 and 23.30 ± 4.74, respectively. The mean scores for assertiveness in the experimental and control groups were 45.53 ± 8.33 and 43.47 ± 9.87, respectively, while the mean scores for social skills were 41.93 ± 6.07 and 40.00 ± 6.89, respectively, for the experimental and control groups. As the groups did not exhibit statistically significant differences for subjective self-esteem, assertiveness, and social skills, the homogeneity of the dependent variables between the two groups was verified (Table 3).

### 3.3. Comparison of Self-Esteem, Self-Expression, and Social Skills between the Groups

The mean scores for subjective self-esteem in the experimental group were 22.90 ± 5.06 and 31.00 ± 4.19 at pre-test and post-test, respectively, whereas the mean scores in the control group were 23.30 ± 4.74 and 23.60 ± 3.98. In other words, the experimental group exhibited increased self-esteem, and this increase significantly differed between the groups (*t* = 6.86, *p* < 0.001). For self-expression, the experimental group had mean scores of 45.53 ± 8.33 and 60.87 ± 6.22 at pre-test and post-test, respectively, while the control group had means of 43.47 ± 9.87 and 45.70 ± 11.76. Thus, self-expression improved in both groups, but this improvement was significantly different (*t* = 8.12, *p* < 0.001) between the groups. Finally, for social skills, the experimental group had mean scores of 41.93 ± 6.07 and 48.77 ± 6.23 at pre-test and post-test, respectively, while the control group had means of 40.00 ± 6.89 and 43.03 ± 7.30. The experimental and control groups both demonstrated increased social skills at post-test. Furthermore, the improvement significantly differed between the groups (*t* = 4.03, *p* < 0.001; Table 4).

## 4. Discussion

This study demonstrated that food art therapy positively affected the self-esteem, self-expression, and social skills of people with mental illness attending community mental rehabilitation facilities. The current finding that food art therapy has a positive effect on participants’ self-esteem is supported by a study [14] in which cooking activities for persons with mental illness result in improved self-reliance and self-esteem. People with mental illness generally have narrow interpersonal relationships and low self-esteem [8], and cooking activities help them experience a sense of accomplishment, have self-confidence, and increase their self-esteem [2]. Another study found that food art therapy increases the self-esteem of female college students without mental disorders [7]. The current study showed that food art and culinary therapy using food ingredients was an intervention that could increase the self-esteem of persons with mental illness.

This study also confirmed that food art therapy positively affected the self-expression ability of persons with mental illness. This finding was consistent with those of previous studies on low-income children [6] and elementary school students from multicultural families [22]. In one study, food art therapy reduces participants’ fear of self-expression because they are able to comfortably eat and touch food in a free atmosphere [6]. Additionally, through a culinary activity program, people are able to freely express their thoughts and opinions, and the finished work gives them joy [23]. A therapeutic cooking program improves self-expression ability, such as the ability to express feelings of love, pleasure, and spiritual satisfaction [24]. Cooking interventions, regardless of whether they are conducted with inpatient or community-based programs, have positively affected participants’ mental health, such as their socialization, self-esteem, quality of life, and mood [1]. Since people with mental illness often lack self-expression abilities and experience difficulties in interacting and establishing relationships with others [15,25], food art therapy can be a useful community-based rehabilitation program for them.

The results of this study showed that food art therapy was effective in improving the social skills of persons with mental illness. This finding was consistent with that of a previous study in which persons with mental problems participate in a group cooking program, and those who have difficulty connecting with others participate comfortably, and their social skills improve [14]. While sharing the finished food, people build a sense of intimacy and trust in the relationship with others and improve their social skills through interaction with others [2,13,26]. The previous studies on New Zealand youth [2], college students with autism [13], and Canadians [26] have also shown that participants’ social skills improve after cooking activity programs.

Social skills have a pivotal impact on interpersonal relationships, social adaptation, and mental health, so social skills improvement is essential for the recovery and rehabilitation of people with mental illness. In particular, group cooking activities, such as food art therapy, develop motor skills and perceptual abilities of participants and support their social and emotional development [27]. In this study, food art therapy had a positive effect on improving the self-esteem, self-expression, and social skills of people with mental illness through the entire process of making food, sharing the food with other people, and clearing it.

This study had a few limitations. First, the participants were from community mental rehabilitation facilities in some regions of South Korea, so caution should be used in generalizing the findings of this study to others living in other regions. Second, participants of this study participated in other activities of the community rehabilitation facilities. The authors could not control the other programs that might have influenced the differences between the experimental and control groups.

## 5. Conclusions

This study confirmed the effectiveness of food art therapy and showed that it could benefit the recovery and rehabilitation of people with mental illness by improving their self-esteem, self-expression, and social skills. Based on this study’s results, it is necessary to implement food art therapy as an intervention program in community mental rehabilitation facilities in other countries. The recovery of people with mental illness includes the rehabilitation in which people return to society and adapt well and live independently. Food art therapy could contribute to the recovery of people with mental illness.

## Figures and Tables

**Table 1:** Overview of eight sessions of food art therapy and examples of the work of participants.

Sessions	Topics	Contents	*Examples of Work*
1	Self-introduction egg sandwich	1. Greeting 2. Pre-test using questionnaires 3. Introduce topic and activities of the day 4. Cooperate with others to make egg sandwiches and express the meaning of their works 5. Wrap-up: eating, clearing	(During the first session, the authors were nervous and forgot to take pictures.)
2	My garden (sweet potato mousse salad)	1. Greeting 2. Introduce topic and activities of the day 3. Make sweet potato mousse together using ingredients to shape ‘my garden’ (non-verbal self-expression) and express the meaning of their works 4. Wrap-up: eating, clearing	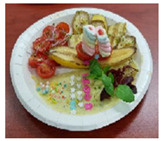
3	The word that I want to hear the most (decorating a pancake)	1. Greeting 2. Introduce topic and activities of the day 3. Decorate a pancake using a chocolate pen and fruits and express the meaning of their works 4. Wrap-up: eating, clearing	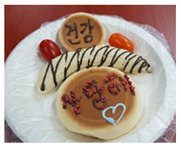
4	My own cupcake	1. Greeting 2. Introduce topic and activities of the day 3. Think about the things that are most valuable and interested in them 4. Express one’s interests using the ingredients provided 5. Wrap-up: eating, clearing	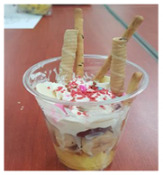
5	Making important people (making rice balls)	1. Greeting 2. Introduce topic and activities of the day 3. Enjoy happy feelings while thinking about important people 4. Create and introduce important people using rice balls 5. Wrap-up: eating, clearing	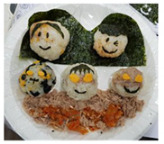
6	My face (my face tortilla)	1. Greeting 2. Introduce topic and activities of the day 3. Consider and introduce one’s strengths 4. Using tortillas, create one’s face and reveal current emotional state and perspective on oneself 5. Wrap-up: eating, clearing	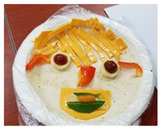
7	A friend’s face (friend face tortilla)	1. Greeting 2. Introduce topic and activities of the day 3. Using tortillas, create the face of a friend and express the friend’s advantages 4. Wrap-up: eating, clearing	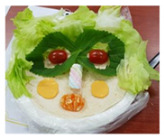
8	Create your own rainbow (fruit skewer)	1. Greeting 2. Introduce topic and activities of the day 3. Have a positive image of the future and introduce one’s dream by making fruit skewers using various fruits 4. Wrap-up: eating, clearing post-test	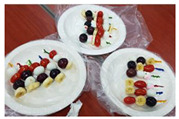

**Table 2:** Homogeneity of demographic characteristics

Demographic Characteristics		Experimental Group (*n* = 30)	Control Group (*n* = 30)	Chi-Square	
				*t*-Test	*p*
Sex	Male	18 (60.0%)	16 (53.3%)	0.27	0.602
	Female	12 (40.0%)	14 (46.7%)		
Marital status	Single	27 (90.0%)	25 (83.3%)	1.21	0.706
	Married	0 (0%)	1 (3.3%)		
	Others	3 (10.0%)	4 (13.3%)		
Age (years)	18–29	6 (20.0%)	3 (10.0%)	1.74	0.627
	30–39	12 (40.0%)	11 (36.7%)		
	40–49	9 (30.0%)	11 (36.7%)		
	50 and over	3 (10.0%)	5 (16.7%)		
Educational level	Middle school or lower	1 (3.3%)	1 (3.3%)	0.31 ^a^	1.000
	High school	25 (83.3%)	25 (83.3%)		
	Bachelor’s degree or higher	4 (13.3%)	4 (13.3%)		
Degree of disability	1	2 (6.7%)	3 (10.0%)	0.48	0.924
	2	1 (3.3%)	1 (3.3%)		
	3	18 (60.0%)	19 (63.3%)		
	None	9 (30.0%)	7 (23.3%)		
Diagnosis	Schizophrenia	27 (90.0%)	28 (93.3%)	0.22	0.640
	Depression	3 (10.0%)	2 (6.7%)		

^a^: Fisher’s exact test results.

**Table 3:** Homogeneity of self-esteem, self-expression, and social skills between the groups.

Variables		Experimental Group (*n* = 30)	Control Group (*n* = 30)	*t*	*p*
		M ± SD	M ± SD		
Self-esteem		22.90 ± 5.06	23.30 ± 4.74	−0.32	0.753
Self-expression	Contents	22.30 ± 2.29	20.97 ± 3.34	1.80	0.076
	Verbal	14.90 ± 4.99	14.33 ± 5.35	0.42	0.673
	Nonverbal	8.33 ± 2.55	8.17 ± 2.61	0.25	0.804
	Total	45.53 ± 8.33	43.47 ± 9.87	0.88	0.385
Social skills	Cooperation	14.30 ± 2.26	13.93 ± 2.42	0.61	0.547
	Assertion	13.63 ± 3.29	12.37 ± 3.83	1.38	0.174
	Self-control	14.00 ± 2.15	13.70 ± 2.29	0.52	0.603
	Total	41.93 ± 6.07	40.00 ± 6.89	1.15	0.254

**Table 4:** Comparison of self-esteem, self-expression, and social skills between the groups.

Variables		Group	Pre-Test	Post-Test	Differences	*t*	*p*
			M ± SD	M ± SD	M ± SD		
Self-esteem		Experimental	22.90 ± 5.06	31.00 ± 4.19	8.10 ± 6.47	6.86	<0.001
		Control	23.30 ± 4.74	23.60 ± 3.98	0.30 ± 7.27		
Self-expression	Contents	Experimental	22.30 ± 2.29	27.07 ± 2.84	4.77 ± 3.31	7.89	<0.001
		Control	20.97 ± 3.34	21.23 ± 4.52	0.27 ± 5.84		
	Verbal	Experimental	14.90 ± 4.99	21.27 ± 2.79	6.37 ± 5.81	6.00	<0.001
		Control	14.33 ± 5.35	15.63 ± 5.68	1.30 ± 7.01		
	Nonverbal	Experimental	8.33 ± 2.55	12.53 ± 1.81	4.20 ± 3.21	7.17	<0.001
		Control	8.17 ± 2.61	8.83 ± 2.99	0.67 ± 3.63		
	Total	Experimental	45.53 ± 8.33	60.87 ± 6.22	15.33 ± 10.34	8.12	<0.001
		Control	43.47 ± 9.87	45.70 ± 11.76	2.23 ± 14.86		
Social skills	Cooperation	Experimental	14.30 ± 2.26	16.43 ± 1.94	2.13 ± 3.45	3.39	0.002
		Control	13.93 ± 2.42	14.60 ± 2.96	0.67 ± 3.06		
	Assertion	Experimental	13.63 ± 3.29	16.20 ± 2.69	2.57 ± 4.65	3.02	0.005
		Control	12.37 ± 3.83	13.77 ± 3.78	1.40 ± 5.38		
	Self-control	Experimental	14.00 ± 2.15	16.13 ± 2.86	2.13 ± 3.35	3.49	0.002
		Control	13.70 ± 2.29	14.67 ± 2.37	0.97 ± 2.77		
	Total	Experimental	41.93 ± 6.07	48.77 ± 6.23	6.83 ± 9.28	4.03	<0.001
		Control	40.00 ± 6.89	43.03 ± 7.30	3.03 ± 9.48		
